# Myeloid derived suppressor and dendritic cell subsets are related to clinical outcome in prostate cancer patients treated with prostate GVAX and ipilimumab

**DOI:** 10.1186/s40425-014-0031-3

**Published:** 2014-09-16

**Authors:** Saskia JAM Santegoets, Anita GM Stam, Sinéad M Lougheed, Helen Gall, Karin Jooss, Natalie Sacks, Kristen Hege, Israel Lowy, Rik J Scheper, Winald R Gerritsen, Alfons JM van den Eertwegh, Tanja D de Gruijl

**Affiliations:** Department of Medical Oncology, VU University Medical Center, Cancer Center Amsterdam, Amsterdam, The Netherlands; Department of Pathology, VU University Medical Center, Cancer Center Amsterdam, Amsterdam, The Netherlands; Cell Genesys Inc, South San Francisco, CA USA; Medarex, Bloomsbury, NJ/Bristol-Myers Squibb Company, Wallingford, CT USA

**Keywords:** Ipilimumab, Prostate GVAX, Biomarker, Patient selection, Survival prediction

## Abstract

**Background:**

Cancer-related disturbances in myeloid lineage development, marked by high levels of myeloid-derived suppressor cells (MDSC) and impaired dendritic cell (DC) development, are associated with poor clinical outcome due to immune escape and therapy resistance. Redressing this balance may therefore be of clinical benefit. Here we investigated the effects of combined Prostate GVAX/ipilimumab immunotherapy on myeloid subsets in peripheral blood of castration-resistant prostate cancer (CRPC) patients as well as the putative predictive value of baseline and on-treatment myeloid parameters on clinical outcome.

**Methods:**

Patients with CRPC (n = 28) received thirteen intradermal administrations of Prostate GVAX, consisting of two allogeneic GM-CSF-transduced and irradiated prostate cancer cell lines (LN-CaP and PC3) and six infusions of escalating doses of anti-CTLA4/ipilimumab. Frequencies and activation status of peripheral blood DC (PBDC) and MDSC were determined before, during and after treatment by flowcytometric analysis and related to clinical benefit.

**Results:**

Significant treatment-induced activation of conventional and plasmacytoid DC subsets (cDC and pDC) was observed, which in the case of BDCA1/CD1c^+^ cDC1 and MDC8^+^/6-sulfoLacNAc^+^ inflammatory cDC3 was associated with significantly prolonged overall survival (OS), but also with the development of autoimmune-related adverse events. High pre-treatment levels of CD14^+^HLA-DR^−^monocytic MDSC (mMDSC) were associated with reduced OS. Unsupervised clustering of these myeloid biomarkers revealed particular survival advantage in a group of patients with high treatment-induced PBDC activation and low pretreatment frequencies of suppressive mMDSC in conjunction with our previously identified lymphoid biomarker of high pretreatment CD4^+^CTLA4^+^ T cell frequencies.

**Conclusions:**

Our data demonstrate that DC and MDSC subsets are affected by prostate GVAX/ipilimumab therapy and that myeloid profiling may contribute to the identification of patients with possible clinical benefit of Prostate GVAX/ipilimumab treatment.

**Electronic supplementary material:**

The online version of this article (doi:10.1186/s40425-014-0031-3) contains supplementary material, which is available to authorized users.

## Background

Prostate cancer is the third leading cause of cancer-related death in men worldwide [1]. Curative treatment options are only available for localized disease. In patients that develop metastatic castration-resistant prostate cancer (mCRPC) the median survival is 16-21 months [2–4]. Recent advances have led to novel immunotherapy options with proven clinical efficacy in patients with metastatic CRPC (mCRPC), such as PROSTVAC and Sipuleucel-T [1,3,4]. In addition, the CTLA-4 blocking antibody ipilimumab has shown clinical activity in a variety of cancer types, including prostate cancer [5–7]. CTLA-4 blockade enhanced antitumor efficacy when combined with other immunomodulating agents, including Granulocyte Macrophage-Colony Stimulating Factor (GM-CSF) and GM-CSF-secreting cancer vaccines, e.g. GVAX immunotherapy [8–10]. In line with this, we recently reported the combined immunotherapy of a GM-CSF-engineered allogeneic tumor cell-based vaccine (Prostate GVAX) and ipilimumab in patients with mCRPC to be safe and clinically active [11]; clinical results included partial responses (PR) and a relatively long survival as compared to survival rates observed in control arms of recent Phase III trials [2–4].

Recent clinical findings have indicated that the induction of an effective antitumor immune response relies on the proper differentiation, maturation and functionality of myeloid antigen-presenting cells (APC), and that the accumulation of myeloid-derived suppressor cells (MDSC) and functionally impaired (immature) dendritic cells (DC) in tumor, blood or lymph nodes of cancer patients is a poor prognostic factor for survival [12–16]. Therefore, cancer immunotherapeutic approaches aiming at the normalization of myeloid differentiation are of interest for clinical application in support of immunotherapy.

We have reported the effects of prostate GVAX/ipilimumab immunotherapy on circulating T cell subsets [17]. However, the net effect of combined prostate GVAX/ipilimumab immunotherapy on DC and MDSC subsets in peripheral blood is currently unknown. It has been described that under proper maturational conditions, peripheral blood DC (PBDC) have the potential to develop into functional DC with the capacity to induce antitumor T-cell responses [18,19]. In particular GM-CSF has been implicated in the recruitment and activation of DC *in vivo* [20,21]. Importantly however, GM-CSF has also been linked to the systemic induction/expansion of MDSC in mice and man [22,23]. Furthermore, CTLA-4 antibody blockade has been shown to reduce MDSC suppressive potency *in vitro*, and *in vivo* in a murine ovarian carcinoma model, and this effect was achieved both indirectly through inhibiting T cell-MDSC interaction [24,25] and directly through binding to CTLA-4 expressed on MDSC [26].

To study whether PBDC and MDSC subsets are affected by prostate GVAX/ipilimumab therapy, extensive myeloid subset monitoring was performed. Myeloid subsets were analyzed prospectively and followed during treatment, after which cut-off points for response to treatment and/or survival were determined in retrospect. Our data demonstrate that PBDC are activated by prostate GVAX/ipilimumab therapy and that a specific myeloid lineage marker profile (i.e. high post-treatment cDC activation and low pre-treatment frequencies of monocytic MDSC) proved predictive for clinical benefit after Prostate GVAX and/or ipilimumab immunotherapy.

## Results

### Clinical results

mCRPC patients (n = 28) received 13 bi-weekly injections of the prostate GVAX vaccine and 6 four-weekly infusions of ipilimumab. As described previously [11], five patients experienced serum-PSA-based PR with PSA declines of more than 50% and 12 demonstrated disease stabilization (SD); PR/SD was significantly correlated with prolonged overall survival (med. survival of 41 versus 21 months; p = 0.0034). Nine patients, all of which received 3 or 5 mg/kg ipilimumab and five of which experienced a PR, developed immune-related adverse events (IRAE) [11]. Interestingly, although IRAE were more frequent in patients that benefited from treatment (i.e. PR and SD; p = 0.0015), the development of IRAE was not associated with survival ([11]; Additional file 1: Figure S1).

### PBDC and monocyte frequency and activation in relation to survival and IRAE

**Figure 1:**
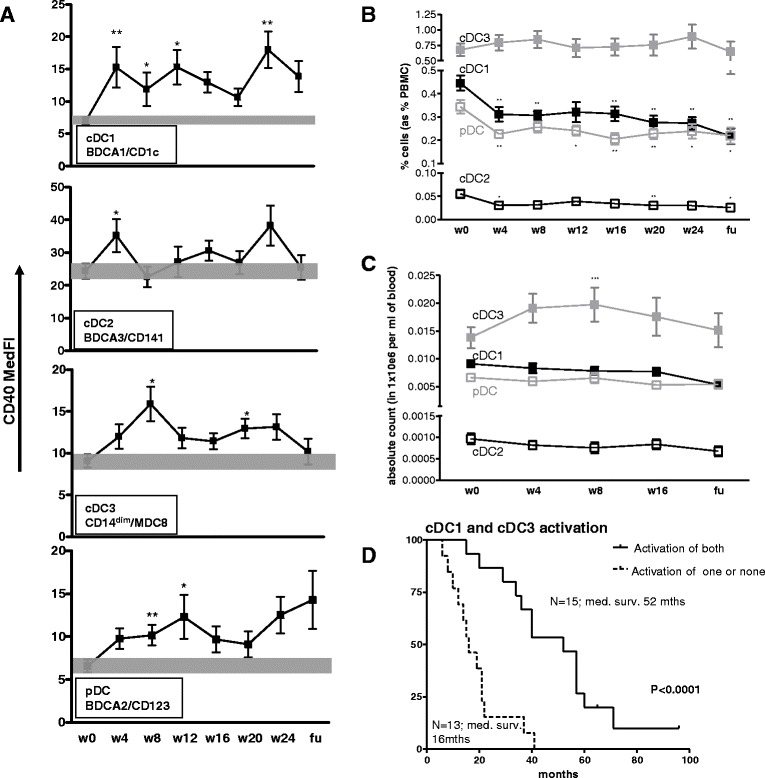
**Prostate GVAX**/**ipilimumab therapy**-**induced activation of peripheral blood** (**PB**) **DC subsets is associated with prolonged survival.** PBDC activation and frequency was determined before (week 0/visit 1 (w0v1)), during (w4v3, w8v5, w12v7, w16v9, w20v11, w24v13 and after (follow-up (fu)) prostate GVAX/ipilimumab therapy by flow cytometry. **A)** Activation state over treatment –by Median Fluorescence Index (MFI) of CD40 of CD11c^hi^CD19^−^CD14^lo^BDCA1^+^cDC1, CD11c^+^CD14^−^BDCA-3^+^ cDC2, CD11c^+^CD14^lo^MDC8^+^ cDC3 and CD11c^−^CD14^−^CD123^hi^BDCA-2^+^pDC. Grey bars denote the mean ± SEM range at baseline. **B)** DC subset frequencies (as percentages of PBMC) and **C)** absolute numbers per ml blood, over treatment, cDC1: solid black squares, cDC2: open black squares, cDC3: solid grey squares, pDC: open grey squares. Means ± SEM of 28 patients are shown. **D)** Kaplan Meier curve for on-treatment increases in cDC1 and cDC3 activation. Number of patients and corresponding median survival for each group is given. Differences between pre- and on- or post-treatment were analyzed with the repeated measures ANOVA with a post-hoc Dunnett’s multiple comparisons test. Differences were considered significant when p < 0.05, as indicated with asterisks (* p < 0.05, ** p < 0.01) within the respective squares. Statistical significance of the survival distribution was analyzed by log-rank testing.

To assess the effects of prostate GVAX/ipilimumab treatment on circulating myeloid DC subsets, frequency and activation status of circulating conventional DC (cDC) subsets cDC1, cDC2, cDC3 and plasmacytoid DC (pDC) were determined before, during and after treatment. cDC1 were identified as CD11c^hi^CD19^−^CD14^−^BDCA-1/CD1c^+^; cDC2 as CD11c^+^CD14^−^BDCA-3^+^; cDC3 as CD11c^hi^CD14^lo^MDC8^+^ and pDC as CD11c^−^CD14^−^CD123^hi^BDCA-2^+^ (see also Additional file 2: Figure S2 for gating strategies). Similar to previous observations in cancer patients by us and by others [12,15,27,28], frequencies and activation status of circulating DC and monocytes were generally lower in CRPC patients as compared with healthy individuals (see Additional file 3: Figure S3A and 3B). On-treatment activation (shown in Figure 1A by CD40 expression levels) was observed for all DC subsets (as previously reported by us for cDC1 [11]). Interestingly, this activation was paralleled by decreases in cDC1, cDC2, and pDC frequencies (Figure 1B). These decreases were observed as early as four weeks after start of treatment and were maintained during treatment (Figure 1B). Of note, decreases in absolute cDC1, CDC2, and pDC numbers per volume blood were much less pronounced and on-treatment increased absolute cDC3 numbers even reached significance (Figure 1C). These differences between DC frequencies and absolute numbers may in large part be explained by a sustained increase in absolute lymphocyte numbers over the course of treatment (see Additional file 4: Figure S4). Increased PBDC activation was generally maintained during treatment (see Figure 1A) and increases of >70% of CD40 med. FI on the cDC1 and cDC3 subsets (see for representative histograms Additional file 2: Figure S2) were associated with significantly prolonged overall survival (OS; median survival 38.5 vs. 15.5 months, p = 0.0004 and median survival 40 vs. 19 months, p = 0.0031, respectively (Table 1). Survival benefit was even more pronounced for patients who displayed treatment-induced activation of *both* cDC1 and cDC3 subsets (median survival 52 vs. 16 months, p < 0.0001; Figure 1C). No relationship with survival was found for either pDC or cDC2.

**Table 1:** **Characteristics and survival distribution of treatment**-**induced changes in PBDC activation and in mMDSC frequencies**

**Immune parameter**	**Mean treatment-induced increase (range)** ^*^	**Cut-off** ^**†**^	**Median survival between groups‡ (# of patients in each group)**	**p-value** ^**§**^	**Hazard ratio (95% CI HR)**	**Mean Halabi Predicted Survival** ^**¶**^	**p-value** ^**׀׀**^
CD40 on cDC1	313% (-67 to +1194)	70%	38.5 vs. 15.5(22 vs. 6)	**0.0004**	0.052 (0.010-0.267)	18.7 vs. 16.6	0.614
CD40 on cDC2	170% (-52 to +612)	150%	36.5 vs. 25.5 (10 vs. 18)	0.397	0.688 (0.299-1.582)	16.8 vs. 19.1	0.338
CD40 on cDC3	160% (-16 to +765)	70%	40.0 vs. 19.0 (19 vs. 9)	**0.0031**	0.179 (0.057-0.560)	18.6 vs. 17.7	0.684
CD40 on pDC	290% (-19 to +1814)	450%	26.0 vs. 31.5 (4 vs. 26)	0.739	1.226 (0.373-4.032)	14.2 vs. 19.0	0.168
CD40 on monocytes	243% (-28 to +1001)	160%	57.0 vs. 21.0 (9 vs. 19)	0.0749	0.469 (0.204-1.079)	20.1 vs. 17.4	0.192
Lin-CD14^+^HLA-DR^−^mMDSC	87% (-71 to +543)	60%	35.0 vs. 36.0 (8 vs. 11)	0.270	0.559 (0.198-1.572)	16.7 vs. 21.4	0.139

^*^Mean and range of treatment-induced increases are given in percentages relative to pre-treatment values.

^†^Cut-off points for survival prediction were determined using the Cox regression model and the relative increments are given as percentage of pre-treatment values.

^‡^Median Survival for both groups was calculated using the Kaplan-Meier method and given in months.

^§^Statistical significance of the survival distribution was analyzed by log-rank testing and considered significant when p < 0.05 (in bold).

^¶^Mean Halabi Predicted Survival (HPS) ± standard error (in months) was determined for patients with biomarker increments above or under designated cut-offs. NB: Halabi scores were determined based on Halabi *et al*. J. Clin. Oncol. 2003 [29], but Halabi scores based on Halabi *et al*. J. Clin. Oncol. 2014 [53] similarly showed prognosis before treatment (based on HPS) not to be the determining factor for any of these myeloid markers in terms of prediction of median survival upon treatment (not shown).

^׀׀^Differences in HPS between groups were determined by Mann-Whitney U test and were considered significant when p < 0.05 (in bold).

**Figure 2:**
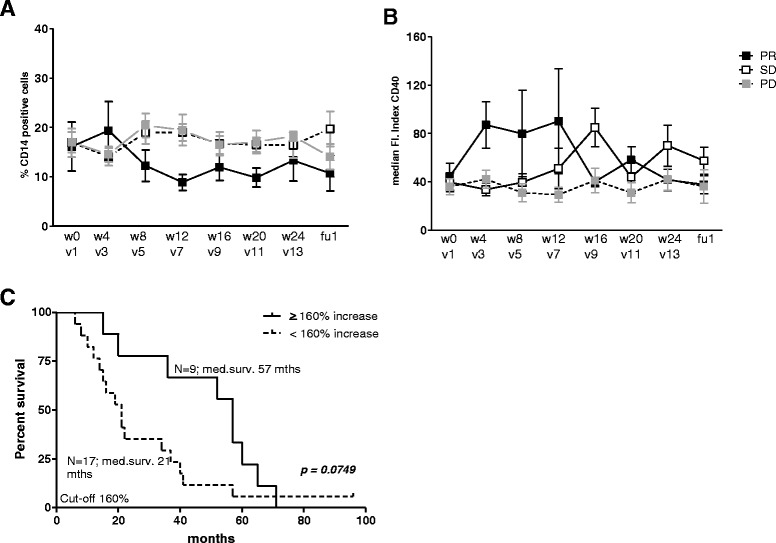
**Increased monocyte activation following Prostate GVAX**/**ipilimumab therapy is associated with prolonged survival.** Frequencies and activation status of circulating CD14^+^ monocytes were determined before (w0), during (w4, w8, w12, w16, w20, w24) and after (fu) Prostate GVAX/ipilimumab treatment by flowcytometric analysis. **A)** Mean percentage (of PBMC) ± SEM and **B)** mean activation ± SEM of CD14+ monocytes is shown before, during and after Prostate GVAX/ipilimumab treatment for 28 patients, divided by clinical PSA response: partial PSA response (PR; black squares), disease stabilization or (SD; white squares) or disease progression (PD; grey squares). Activation is given as med. Fluorescence Index (med. FI) and calculated by dividing the med. fluorescence (med. fl) of CD40 antibody by the med. fl of the isotype control antibody. **C)** Kaplan Meier curve for treatment-induced increases in activation of monocytes. Number of patients and corresponding median survival for each group are given. Differences between pre- and on- or post-treatment were analyzed with the repeated measures ANOVA with a post-hoc Dunnett’s multiple comparisons test. Differences were considered significant when p < 0.05, as indicated with asterisks (* p < 0.05, ** p < 0.01).

When patients were divided by treatment response, decreases in the frequency of monocytes were found to be selectively associated with PR (Figure 2A), which, like observed for PBDC subsets, was paralleled by increased activation (Figure 2B). Of note, although these were clear trends, they did not reach statistical significance. Similarly to the cDC1/BDCA1 and cDC3/SLAN-DC subsets, treatment-induced increases of CD40 med. FI on CD14^+^ monocytes was associated with prolonged OS (median survival 57 vs. 21 months, p = 0.0749; Figure 2C and Table 1).

**Figure 3:**
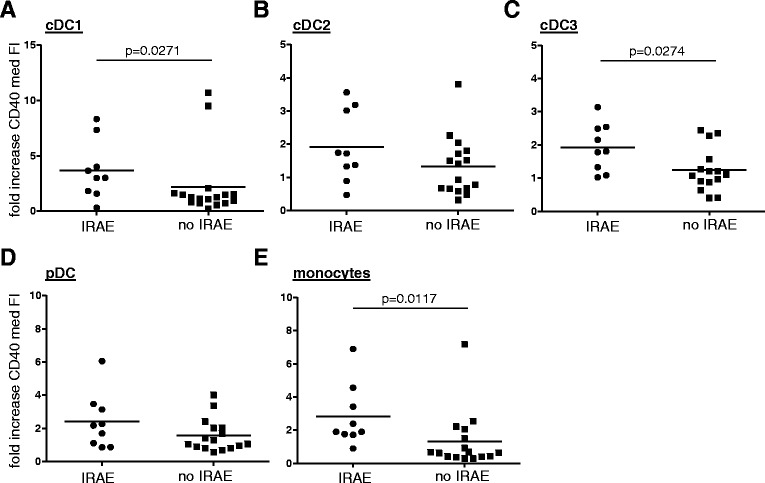
**PBDC activation in relation to immune**-**related adverse events (IRAE).** Fold increase in activation of **A)** cDC1, **B)** cDC2, **C)** cDC3, **D)** pDC and **E)** monocytes was determined at week 4 after start of prostate GVAX/ipilimumab treatment by dividing the median Fluorescence Index (med. FI) of CD40 at week four (i.e. 2 vaccinations and 1 ipilimumab infusion) by the med. FI of CD40 at start of treatment and displayed for patients that experienced IRAE or no IRAE during therapy. Differences in fold increase in activation between groups of patients were analyzed with the two-sample Mann-Whitney U test. Differences were considered significant when p < 0.05, as indicated with the given p-value.

To determine whether changes in DC and monocyte activation status could possibly serve as early marker for the ipilimumab-associated development of IRAE, we compared the rise in DC or monocyte activation status at week four of treatment (i.e. after two GVAX administrations and only one ipilimumab infusion) between patients with or without IRAE. As shown in Figure 3, treatment-induced increases in cDC1, cDC3, and monocyte activation were significantly higher in patients that eventually developed IRAE, suggesting that these treatment-induced increases might serve as an indicator for risk of IRAE.

### MDSC frequencies in relation to survival

**Figure 4:**
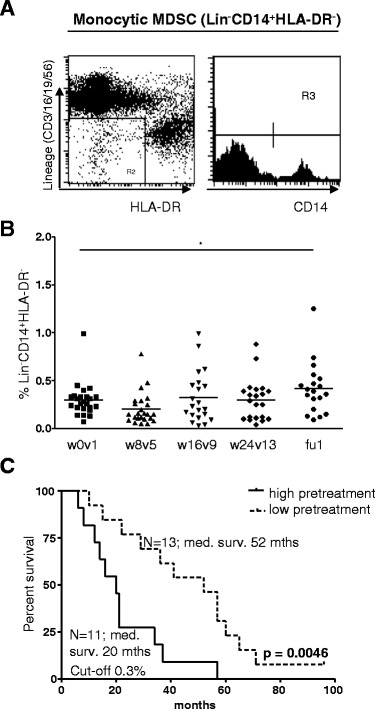
**High pretreatment frequency of mMDSC is associated with reduced OS.** mMDSC frequencies were determined before (week 0/visit 1 (w0v1)), during (w8v5, w16v9 and w24v13) and after (follow-up (fu)) prostate GVAX/ipilimumab therapy by flow cytometry. **A)** A representative analysis of Lin^−^CD14^+^HLA-DR^−/lo^ mMDSC. **B)** Percentage of mMDSC over follow-up. **C)** Kaplan meier curve for pre-treatment frequencies of mMDSC. Number of patients and corresponding median survival for each group are given. Differences between pre- and on- or post-treatment were analyzed with the repeated measures ANOVA with a post-hoc Dunnett’s multiple comparisons test. Differences were considered significant when p < 0.05, as indicated with asterisks (* p < 0.05, ** p < 0.01). Statistical significance of the survival distribution was analyzed by log-rank testing and indicated with the given p-value.

To assess the effects of prostate GVAX/ipilimumab treatment on circulating MDSC, the frequency of monocytoid MDSC (mMDSC; Lin^־^CD14^+^HLA-DR^־/lo^, see Figure 4A) was determined. Significantly higher levels of circulating mMDSC were detected in CRPC patients compared with age- and sex-matched healthy individuals. Significant post-treatment increases in mMDSC frequencies were observed (Figure 4B), yet these increases were only modest and did not correlate with response to treatment (not shown) or survival (Table 1). In contrast, patients who displayed high pre-treatment levels of mMDSC had a significantly shorter OS than patients who did not (Figure 4C, median survival 20 vs. 52 months, HR = 4.26, 95% CI = 1.37 - 13.25, p = 0.0046).

### A myeloid marker profile of high DC activation and low frequencies of MDSC is predictive for survival

To assess whether clinical prognosis impacted the putative predictive value for treatment outcome of any of the identified myeloid parameters, the median Halabi Predicted Survival (HPS) was determined for the patient groups above and below the designated cut-offs [29]. No significant differences were observed, indicating that better prognosis before treatment was not the determining factor for any of these parameters (Table 1).

**Figure 5:**
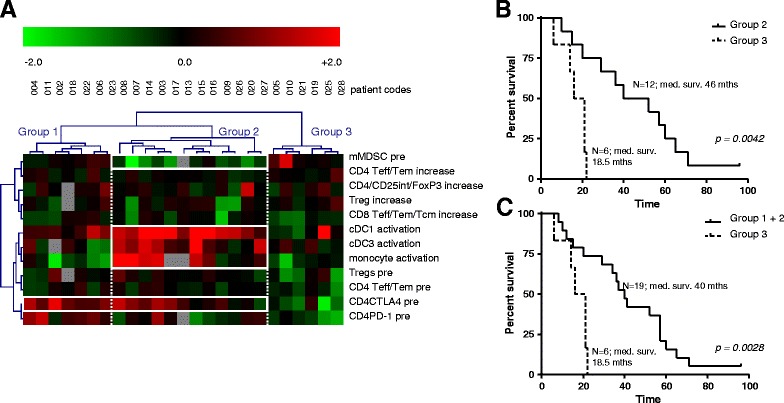
**High on**-**treatment cDC1/cDC3/monocyte activation and low pretreatment mMDSC frequencies predict clinical benefit after therapy. A)** Unsupervised cluster analysis of the expression of the indicated treatment-induced and pre-treatment myeloid and lymphoid markers. To identify clusters of correlated markers, hierarchical cluster analysis using TIGR software was performed, and average linkage analysis was done by Pearson correlation analysis. Values of the treatment-induced and pre-treatment parameters are given relative to the cut-off value (determined by Cox regression model as described in materials and methods); below cut-off in green and above cut-off in red. Kaplan Meier curves for **B)** group 2 (with high on-treatment cDC1/cDC3/monocyte activation and low pretreatment mMDSC frequencies: highlighted by white boxes in the heat plot) versus group 3 (with generally low on-treatment cDC1/cDC3/monocyte activation and high mMDSC frequencies) and **C)** group 1 + 2 (with high pre-treatment frequencies of CD4^+^CTLA4^+^ T cells: lower white box in heat plot) versus group 3. Statistical significance of the survival distribution was analyzed by log-rank testing and indicated with the given p-value. Number of patients and corresponding median survival for each group are given.

We previously identified a lymphoid biomarker profile with predictive value for OS benefit, which was dominated by high CD4^+^CTLA4^+^ T cell frequencies prior to therapy [17]. When combining all predictive lymphoid [17] and myeloid markers in an unsupervised cluster analysis, we found a particularly strong association with prolonged survival for patients displaying a combination of high levels of on-treatment cDC1/cDC3/monocyte activation, low pretreatment mMDSC rates and high pretreatment frequencies of CD4^+^CTLA4^+^ T cells (designated clustered group 2, median OS 46 months, see Figure 5A and 5B). Nevertheless, patients with relatively high pretreatment frequencies of CD4^+^CTLA4^+^ T cells showed significant survival benefit whether these were accompanied by concerted high levels of on-treatment cDC1/cDC3/monocyte activation and low pretreatment mMDSC rates or not (designated clustered groups 2 + 1, median OS 40 months; see Figure 5A and 5C).

## Discussion

PBDC and MDSC profiling of patients with CRPC receiving combined Prostate GVAX/ipilimumab treatment revealed that PBDC and MDSC subsets were affected by prostate GVAX/ipilimumab therapy, with on-treatment increases in DC activation and mMDSC frequencies. Of note, a myeloid profile of low pretreatment frequencies of mMDSC and high treatment-induced cDC1, cDC3, and monocyte activation levels demonstrated predictive value for OS on treatment. As this was only a small exploratory study of 28 patients, no multivariate analyses were performed and the candidate biomarkers should be validated prospectively in larger randomized trials.

It has been reported that frequencies and activation status of circulating DC are significantly lower in cancer patients compared with healthy individuals [12,15,27,28]. In our study, similar results were observed, with significantly lower cDC1 frequencies and an inferior activation state of cDC1, cDC2 and monocytes in mCRPC patients (see Additional file 3: Figure S3A and *B*). Interestingly, prostate GVAX/ipilimumab treatment resulted in a further reduction of circulating cDC and pDC subsets, and this reduction was paralleled by increases in their activation status. The observed activation may be explained by the production of GM-CSF by the vaccine, as GM-CSF has been described to activate DC *in vivo* [20,21]. Alternatively, or additionally, ipilimumab may have altered DC activation state through blocking CTLA-4/B7 interactions at the Treg/DC interface or by direct binding of CTLA-4 on DC [30–32]. Nevertheless, a lack of association between ipilimumab dose and DC activation seems to support an overriding role for GVAX-derived GM-CSF in this respect. The enhanced activation and simultaneous reduction of cDC and pDC subset frequencies in blood is suggestive of their recruitment to effector sites (e.g. tumor and vaccination sites). This hypothesis is further supported by our own observation of enhanced recruitment of antigen-presenting cells to the Prostate GVAX vaccination sites following repeated vaccination (Van Mens *et al*, manuscript in preparation). However, absolute PBDC subset numbers showed more moderate decreases whereas they revealed a significant on-treatment increase in cDC3. The latter fits well with the inflammatory nature of cDC3 or SLAN-DC, being mobilized by cytokines like GM-CSF [33].

MDSC represent a heterogeneous population of immature myeloid cells, and have been recognized to play an important role in suppression of (anti-tumor) immune responses. Elevated levels of either monocytoid or granulocytic MDSC have been observed in a variety of human cancers [34–38]. Moreover, their presence in peripheral blood or at the tumor site has been linked with poor prognosis and may thus serve as a prognostic or predictive marker for clinical outcome [36,39–41]. In keeping with previous findings, mCRPC patients displayed significantly higher levels of mMDSC compared with age- and sex-matched healthy controls (see Additional file 3: Figure S3*C*). In fact, significantly prolonged OS was observed for patients displaying low pre-treatment levels of mMDSC, i.e. levels that were similar to those found in healthy volunteers, suggesting that indeed mMDSC levels may serve as a predictive marker for clinical outcome. This is in keeping with a recent report indicating a similar predictive value of pre-treatment mMDSC frequencies for outcome of tumor peptide vaccination therapy in patients with renal cell cancer [39].

Our data demonstrated modest increases of mMDSC frequencies following multiple Prostate GVAX/ipilimumab doses in a subgroup of patients. As described, increases in the frequency of circulating MDSC can be related to progression [39,42–44]. No such relation was observed in our study, as no difference in mMDSC expansion was observed between PR, SD and PD patients (not shown). The observed mMDSC expansion might have been induced by the Prostate GVAX vaccine, since GM-CSF-based vaccines have been shown to expand MDSC in mice and man [22,23]. Perhaps surprisingly, the post-vaccination increases of circulating mMDSC frequencies did not impact survival in our study, suggesting that prostate GVAX/ipilimumab therapy may reduce the suppressive function of mMDSC. Indeed, reduction of MDSC suppressive function has been described for CTLA-4 blockade therapy in an *in vitro* and *in vivo* murine ovarian carcinoma model [24–26]. Moreover, as mMDSC have been shown to further develop into more mature macrophage and DC-like cells [42], GM-CSF-driven differentiation may have interfered with their suppressive ability.

A major problem in CTLA-4 blockade therapy is the development of potentially life-threatening IRAE like colitis, hepatitis, alveolitis and hypophysitis [45,46]. To date, attempts to reduce the development of these IRAE have been unsuccessful [47]. Therefore, it is important to identify biomarkers for patient selection or develop methods that enable the early detection of IRAE. Within the myeloid compartment, no pre-treatment marker with putative clinical value for IRAE risk prediction could be identified. Yet, our data showed that the magnitude of early (i.e. at week 4, after only one single ipilimumab administration) CD40 up-regulation on cDC1, cDC3 and monocytes correlated with on-treatment IRAE development. Unfortunately, the clinical applicability of this on-treatment change as a marker for IRAE risk is questionable, as there is considerable overlap for CD40 activation levels on the indicated subsets between the IRAE positive and negative group (Figure 3).

Our profiling data are consistent with clinical benefit and survival advantage for patients with high DC activation and low levels of MDSC. These data echo our finding of a predictive T cell profile of activated T cells and low pre-treatment Treg frequencies in the same group of patients [17], and are in line with an ever growing number of studies stressing the importance of a pro-inflammatory, non-suppressive immune status for optimal efficacy of immunotherapy in cancer patients. Interestingly, our unsupervised clustering analysis of combined myeloid and lymphoid biomarkers indicates two, partially overlapping, major populations with survival benefit: 1) patients with combined low pre-treatment frequencies of mMDSC and high on-treatment cDC activation levels, and 2) patients with high pre-treatment levels of CD4^+^CTLA4^+^ T cells, see Figure 5A, white-lined boxes. Whereas the latter biomarker profile may signal particular susceptibility to check point inhibition [17], the first may be representative of a favorable immune state and as such hold predictive value for immunotherapy in general. These profiles warrant further assessment and validation of their utility for patient selection in other, preferably randomized, clinical trials of GM-CSF-based therapy and/or CTLA-4 blockade or other forms of cancer immunotherapy.

## Conclusions

This study provides evidence that circulating myeloid subsets are affected by combined Prostate GVAX and ipilimumab therapy and that a myeloid lineage profile of low pretreatment mMDSC frequencies and high treatment-induced cDC activation levels may contribute to the identification of patients with possible clinical benefit of Prostate GVAX/ipilimumab treatment.

## Methods

### Prostate GVAX and ipilimumab

The Prostate GVAX vaccine is a cellular vaccine consisting of two prostate cancer cell lines, LNCaP (CG8711) and PC-3 (CG1940), which have been transduced with an adeno-associated viral vector to secrete GM-CSF. These cell lines were propagated, frozen, and irradiated to arrest further cell division [6,48]. The product was stored and shipped in gaseous nitrogen phase, and administered within 60 minutes after thawing. All manufacturing was conducted according to good manufacturing practice. Ipilimumab (formerly MDX-010), a fully human IgG1κ monoclonal antibody directed against CTLA-4, was provided by Medarex/Bristol-Myers Squibb (Plainsboro, NJ, USA).

### Study population and sampling of peripheral blood

As described [11], 28 chemonaive patients with asymptomatic mCRPC received 13 bi-weekly vaccinations of the Prostate GVAX vaccine and 6 four-weekly infusions of ipilimumab from the time of prime vaccination. In the first 12 patients, ipilimumab was administered at escalating doses of 0.3, 1, 3 and 5 mg/kg (3 patients each). In the expansion phase, 16 additional patients were included at 3 mg/kg ipilimumab. This study is registered with the Central Committee on Research involving Human Subjects in the Netherlands, number P03.1786C, and ClinicalTrials.gov, number NCT01510288.

Responses to treatment were defined as described [49]. In brief, PSA partial response (PR) was defined as >50% PSA decline from baseline, which was confirmed by a second PSA test 3 or more weeks later. PSA progressive disease (PD) was defined as >25% PSA increase and an absolute increase of 2 ng/ml or more from baseline, whereas stable disease (SD) was defined as no PR and no PD on treatment.

For immune monitoring, blood samples were taken from the patients before start of therapy and every four weeks thereafter until four weeks after the last treatment (i.e. follow-up (fu)). Peripheral blood mononuclear cells (PBMC) were isolated by density centrifugation (NycomedAS, Oslo, Norway). PBMC were either directly used for PBDC or monocyte analysis or cryopreserved for later MDSC flow cytometric analysis.

### Antibodies and 4-color flow cytometry

PBDC and MDSC frequencies and activation status were assessed before, during and after treatment by flow cytometry staining as described [17]. Cell surface antibody staining of PBMC was performed in PBS/0.1% BSA/0.02% Sodium-Azide (hereafter referred to as FACS buffer) for 30 minutes at 4°C. The following antibodies were used: fluorescein isothiocyanate- (FITC), phycoerythrin- (PE), peridinin chlorophyll protein-Cy5.5- (PerCP) or allophycocyanin (APC)-labeled Abs directed against human CD3, CD11c, CD14, CD16, CD19, CD33, CD56, CD123 and HLA-DR (all BD Bioscience), CD40 (Beckman Coulter, Marseille, France), Fab-M-FITC (Southern Biotec, Birmingham, AL) and blood DC antigens BDCA1, BDCA2, BDCA3 (all from Milteny Biotec, BergischGladbach, Germany) and MDC8 (a kind gift from Dr. E.P. Rieber, Dresden, Germany) and matching isotype control antibodies. Stained cells were analyzed on aFACScalibur (BD Biosciences) using Cell Quest software. Events collected were 120,000-150,000 per sample.

### PBDC and MDSC subset and activation definitions

PBDC frequencies were determined on the basis of expression of BDCA or MDC8 markers: two major myeloid or conventional DC (cDC) subsets as recognized by the Nomenclature Committee of the International Union of Immunological Societies were identified as CD11c^hi^CD19^−^CD14^−^BDCA-1/CD1c^+^ DC (designated cDC1) and as CD11c^+^CD14^−^BDCA-3^+^ DC (designated cDC2) [50,51]; in addition we assessed frequencies of a third myeloid subset, designated cDC3, and defined as CD11c^+^CD14^lo^MDC8^+^ DC (also known as 6-sulfo LacNAc^+^ non-classical monocytes or SLAN-DC [33,52]) as well as plasmacytoid DC (pDC), defined as CD11c^−^CD14^−^CD123^hi^BDCA-2^+^ [28]. Classic monocytes were defined as CD11c^hi^CD14^hi^ (highly positive; see also Additional file 2: Figure S2). Monocytoid MDSC (mMDSC) were defined as Lin^−^CD14^+^HLA-DR^neg/lo^ [28] (see also Figure 4A). For all the above mentioned populations a live gate was used based on FSC-SSC properties of the lymphocyte and monocyte populations. Activation status of above mentioned cDC, pDC and monocyte subsets was determined by calculating the median Fluorescence Index (med. FI) of CD40 expression by dividing the med. fluorescence (Med. fl) of the CD40 antibody by the med. fl of the isotype-control antibody.

### Statistical analyses

Differences between immune parameters before treatment (w0v1; w = week; v = visit; i.e. baseline levels) and during and/or after treatment (w4v3, w8v5, w12v7, w16v9, w20v11, w24v13 and follow up [fu, i.e. 1 month after last Prostate GVAX and 2 months after last ipilimumab administration]) were analyzed with the repeated measures ANOVA with a post-hoc Dunnett’s multiple comparisons test. To determine whether the identified immune parameters were indicative for response to treatment or useful for survival prediction, optimal cut-off points were determined by Cox regression analysis, according to which patients were subsequently divided into two groups. OS for the two groups was plotted using the Kaplan-Meier method and statistical significance of the survival distribution was analyzed by log-rank testing. To analyze whether prognosis impacted the value of the identified response/survival parameters, the median Halabi Predicted Survival (HPS) was also determined for both groups ([29,53]; see also Table 1). Differences in HPS between groups of patients and in parameters between prostate cancer patients and age and sex-matched healthy volunteers were analyzed with the two-sample Mann-Whitney U or the Fisher’s exact test (both two-tailed). Above listed statistical analyses were performed either with Prism GraphPad or SPSS software. Differences were considered significant when p < 0.05. To identify clusters of correlated markers, hierarchical cluster analysis using TIGR software was performed and complete linkage analysis was done by Pearson correlation analysis. For this purpose, the values of the treatment-induced and pre-treatment parameters were taken for each patient, and divided by the cut-off value, after which the resulting ratios were log-transformed (base 2). Three patients were excluded from this analysis since <70% of the analyzed biomarkers were available for these patients due to withdrawal from the study or sampling failure.

## Additional files

Additional file 1: Figure S1. IRAE in relation to treatment response and survival. **A)** Distribution of IRAE within the different treatment response groups (i.e. partial response (PR), Stable Disease (SD) and Progressive Disease (PD)) is given. Black bars: patients experiencing IRAE and white/open bars: patients without IRAE during treatment. **B)** Kaplan Meier curve for the patients that experienced IRAE. Number of patients and corresponding median survival for each group are given. Differences in distribution of IRAE between treatment response groups were analyzed with a two-tailed Fisher’s exact test. Statistical significance of the survival distribution was analyzed by log-rank testing. Differences were considered significant when p < 0.05. Additional file 2: Figure S2. Peripheral Blood DC (PBDC) gating strategy. **A)** First, live cells were gated based on FSC-SSC properties of the lymphocyte and monocyte populations (not shown), after which the cDC1, cDC2, cDC3 and pDC populations were identified through BDCA1 (and CD19^−^, not shown), BDCA3, MDC8 and BDCA2 expression. By backgating on CD11c and CD14 as indicated, the identity of the different DC subsets was confirmed. Activation status of abovementioned cDC, pDC and monocyte subsets was determined by calculating the median Fluorescence Index (med. FI) of CD40 expression by dividing the med. fluorescence (Med. fl) of the CD40 antibody by the med. fl of the isotype-control antibody. **B)** Isotype control and CD40 histograms are depicted for the cDC1 (left) and cDC3 (right) subsets at week (w) 0 visit (v) 1 (i.e. baseline) and w4v3 for a representative patient. Additional file 3: Figure S3. PBDC frequencies and activation status and MDSC frequencies in mCRPC patients and healthy individuals. Frequencies of cDC, pDC, monocytes and gMDSC and mMDSC subsets, and activation status of cDC, pDC and monocytes was determined in mCRPC patients before prostate GVAX/ipilimumab therapy and age- and sex-matched healthy donors (HD). Percentage **A**) and activation status **B**) of cDC1, cDC2, cDC3, pDC and monocytes and percentage of **C**) mMDSC are shown. Differences in percentage or activation between mCRPC and HD were analyzed with the two-sample Mann-Whitney U test. Differences were considered significant when p < 0.05, as indicated with the given p-value. Additional file 4: Figure S4. Differential leukocyte analysis in mCRPC patients before and during prostate GVAX/ipilimumab therapy. Absolute lymphocytes (white squares) and monocyte (black squares) counts were determined before (week 0/visit 1 (w0v1)), during (w4v3, w8v5, w16v9) and after (follow-up (fu)) prostate GVAX/ipilimumab therapy. Mean absolute counts ± SEM is given in 10^e^6 per ml of blood for lymphocytes (white squares), monocytes (black squares) and the sum of lymphocytes and monocytes (i.e. PBMC, grey squares). Differences between pre- and on- or post-treatment were analyzed with the repeated measures ANOVA with a post-hoc Dunnett’s multiple comparisons test. Differences were considered significant when p < 0.05, as indicated with an asterisk (* p < 0.05, ** p < 0.01).

## Abbreviations

cDC: Conventional dendritic cell
CTLA-4: CTL antigen-4
CRCP: Castration resistant prostate cancer
fu: Follow-up
HPS: Halabi predicted survival
IRAE: Immune related adverse events
MDSC: Myeloid-derived suppressor cell
OS: Overall survival
pDC: Plasmacytoid dendritic cell
PD: Progressive disease
PR: Partial response
SD: Stable disease
