# Supplementary material for: Myeloid derived suppressor and dendritic cell subsets are related to clinical outcome in prostate cancer patients treated with prostate GVAX and ipilimumab
Source: J Immunother Cancer. 2014 Sep 16;2:31. doi: 10.1186/s40425-014-0031-3 (PMC4507359; doi:10.1186/s40425-014-0031-3)
Supplement: Additional file 1: Figure S1. — IRAE in relation to treatment response and survival. A) Distribution of IRAE within the different treatment response groups (i.e. partial response (PR), Stable Disease (SD) and Progressive Disease (PD)) is given. Black bars: patients experiencing IRAE and white/open bars: patients without IRAE during treatment. B) Kaplan Meier curve for the patients that experienced IRAE. Number of patients and corresponding median survival for each group are given. Differences in distribution of IRAE between treatment response groups were analyzed with a two-tailed Fisher’s exact test. Statistical significance of the survival distribution was analyzed by log-rank testing. Differences were considered significant when p < 0.05. [file 40425_2014_31_MOESM1_ESM.ppt]

## Slide 1
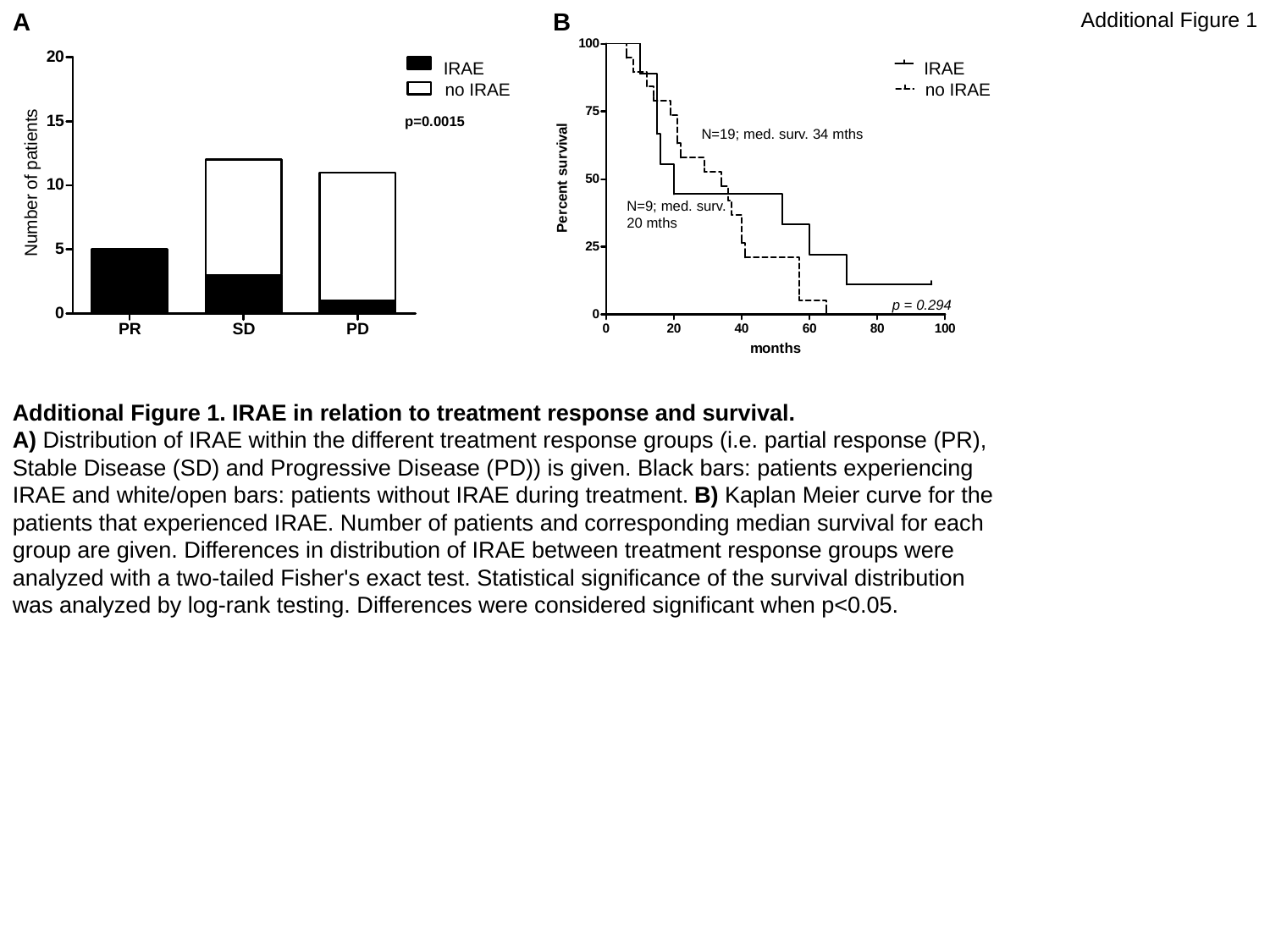

A
B
Additional Figure 1
IRAE
IRAE
no IRAE
no IRAE
p=0.0015
N=19; med. surv. 34 mths
Number of patients
N=9; med. surv.
20 mths
p = 0.294
Additional Figure 1. IRAE in relation to treatment response and survival.
A) Distribution of IRAE within the different treatment response groups (i.e. partial response (PR), Stable Disease (SD) and Progressive Disease (PD)) is given. Black bars: patients experiencing IRAE and white/open bars: patients without IRAE during treatment. B) Kaplan Meier curve for the patients that experienced IRAE. Number of patients and corresponding median survival for each group are given. Differences in distribution of IRAE between treatment response groups were analyzed with a two-tailed Fisher's exact test. Statistical significance of the survival distribution was analyzed by log-rank testing. Differences were considered significant when p<0.05.
