# Supplementary material for: Myeloid derived suppressor and dendritic cell subsets are related to clinical outcome in prostate cancer patients treated with prostate GVAX and ipilimumab
Source: J Immunother Cancer. 2014 Sep 16;2:31. doi: 10.1186/s40425-014-0031-3 (PMC4507359; doi:10.1186/s40425-014-0031-3)
Supplement: Additional file 2: Figure S2. — Peripheral Blood DC (PBDC) gating strategy. A) First, live cells were gated based on FSC-SSC properties of the lymphocyte and monocyte populations (not shown), after which the cDC1, cDC2, cDC3 and pDC populations were identified through BDCA1 (and CD19−, not shown), BDCA3, MDC8 and BDCA2 expression. By backgating on CD11c and CD14 as indicated, the identity of the different DC subsets was confirmed. Activation status of abovementioned cDC, pDC and monocyte subsets was determined by calculating the median Fluorescence Index (med. FI) of CD40 expression by dividing the med. fluorescence (Med. fl) of the CD40 antibody by the med. fl of the isotype-control antibody. B) Isotype control and CD40 histograms are depicted for the cDC1 (left) and cDC3 (right) subsets at week (w) 0 visit (v) 1 (i.e. baseline) and w4v3 for a representative patient. [file 40425_2014_31_MOESM2_ESM.ppt]

## Slide 1
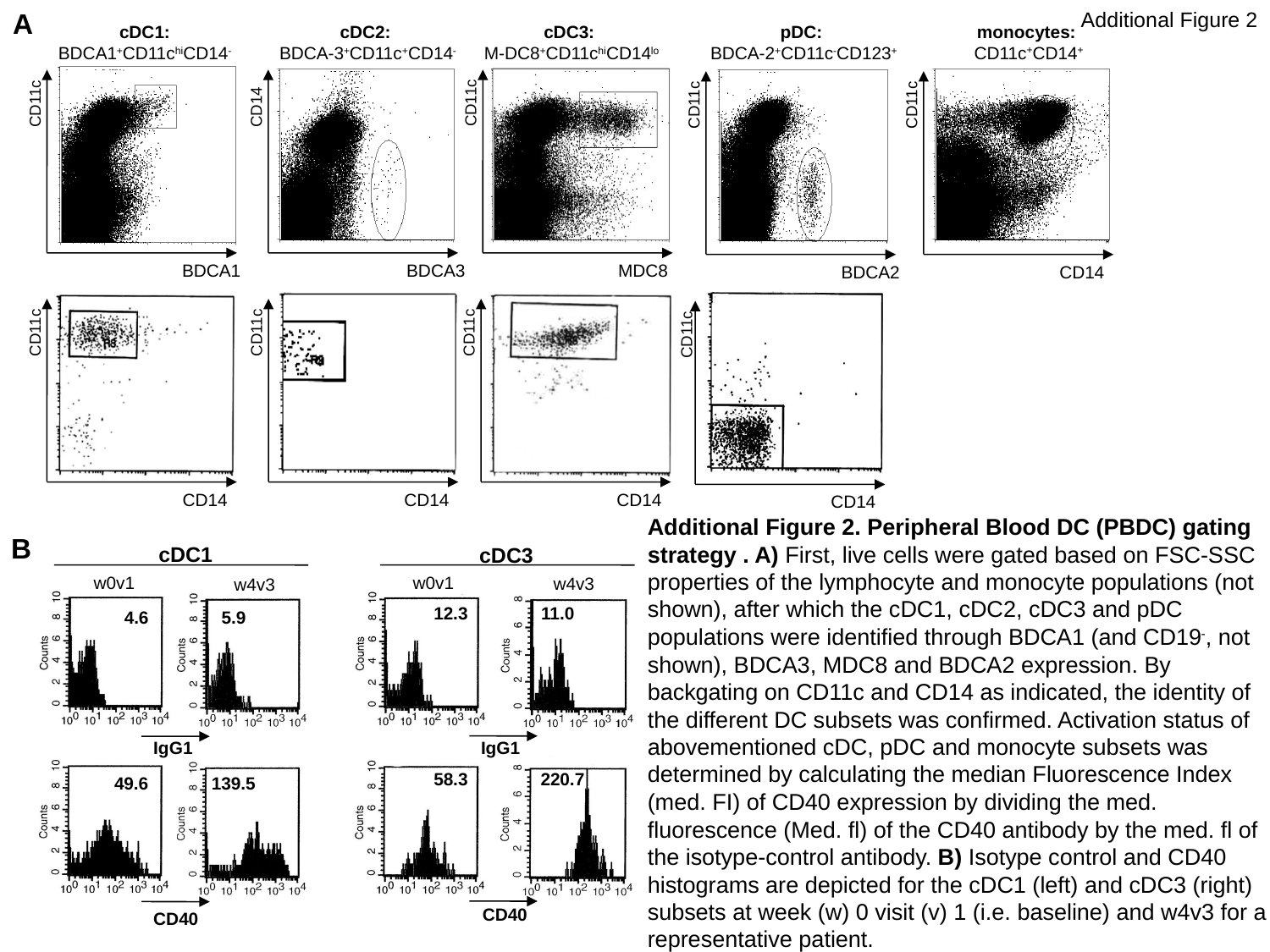

A
Additional Figure 2
monocytes:
CD11c+CD14+
cDC1:
BDCA1+CD11chiCD14-
cDC2:
BDCA-3+CD11c+CD14-
cDC3:
M-DC8+CD11chiCD14lo
pDC:
BDCA-2+CD11c-CD123+
CD11c
CD11c
CD11c
CD11c
CD14
BDCA1
BDCA3
MDC8
BDCA2
CD14
CD11c
CD11c
CD11c
CD11c
CD14
CD14
CD14
CD14
B
Additional Figure 2. Peripheral Blood DC (PBDC) gating strategy . A) First, live cells were gated based on FSC-SSC properties of the lymphocyte and monocyte populations (not shown), after which the cDC1, cDC2, cDC3 and pDC populations were identified through BDCA1 (and CD19-, not shown), BDCA3, MDC8 and BDCA2 expression. By backgating on CD11c and CD14 as indicated, the identity of the different DC subsets was confirmed. Activation status of abovementioned cDC, pDC and monocyte subsets was determined by calculating the median Fluorescence Index (med. FI) of CD40 expression by dividing the med. fluorescence (Med. fl) of the CD40 antibody by the med. fl of the isotype-control antibody. B) Isotype control and CD40 histograms are depicted for the cDC1 (left) and cDC3 (right) subsets at week (w) 0 visit (v) 1 (i.e. baseline) and w4v3 for a representative patient.
cDC1
w0v1
w4v3
4.6
5.9
IgG1
49.6
139.5
CD40
cDC3
w0v1
w4v3
12.3
11.0
IgG1
58.3
220.7
CD40
