# Supplementary material for: Myeloid derived suppressor and dendritic cell subsets are related to clinical outcome in prostate cancer patients treated with prostate GVAX and ipilimumab
Source: J Immunother Cancer. 2014 Sep 16;2:31. doi: 10.1186/s40425-014-0031-3 (PMC4507359; doi:10.1186/s40425-014-0031-3)
Supplement: Additional file 3: Figure S3. — PBDC frequencies and activation status and MDSC frequencies in mCRPC patients and healthy individuals. Frequencies of cDC, pDC, monocytes and gMDSC and mMDSC subsets, and activation status of cDC, pDC and monocytes was determined in mCRPC patients before prostate GVAX/ipilimumab therapy and age- and sex-matched healthy donors (HD). Percentage A) and activation status B) of cDC1, cDC2, cDC3, pDC and monocytes and percentage of C) mMDSC are shown. Differences in percentage or activation between mCRPC and HD were analyzed with the two-sample Mann-Whitney U test. Differences were considered significant when p < 0.05, as indicated with the given p-value. [file 40425_2014_31_MOESM3_ESM.ppt]

## Slide 1
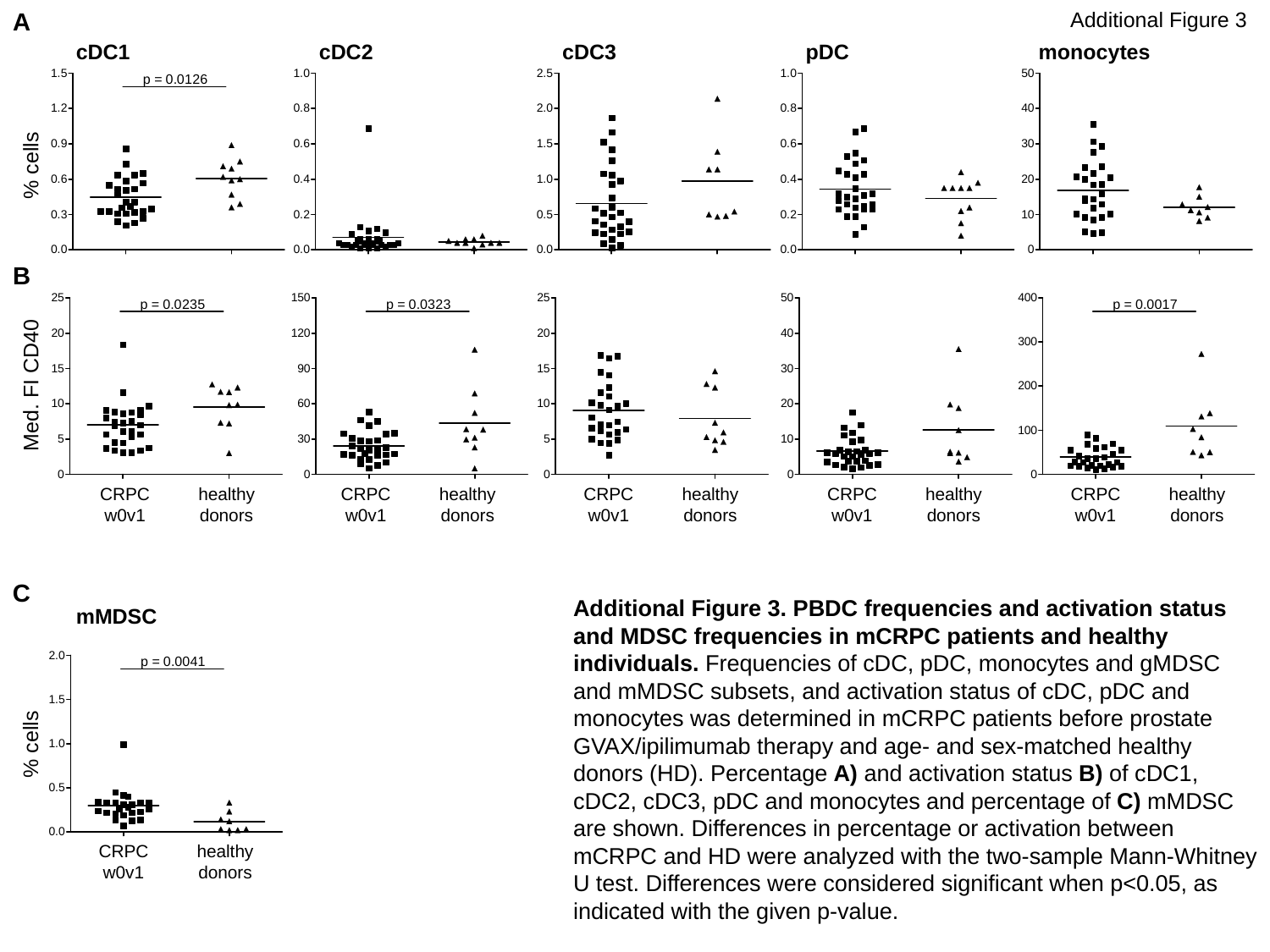

A
Additional Figure 3
cDC1
cDC2
cDC3
pDC
monocytes
% cells
B
Med. FI CD40
CRPC
w0v1
healthy
donors
CRPC
w0v1
healthy
donors
CRPC
w0v1
healthy
donors
CRPC
w0v1
healthy
donors
CRPC
w0v1
healthy
donors
C
Additional Figure 3. PBDC frequencies and activation status and MDSC frequencies in mCRPC patients and healthy individuals. Frequencies of cDC, pDC, monocytes and gMDSC and mMDSC subsets, and activation status of cDC, pDC and monocytes was determined in mCRPC patients before prostate GVAX/ipilimumab therapy and age- and sex-matched healthy donors (HD). Percentage A) and activation status B) of cDC1, cDC2, cDC3, pDC and monocytes and percentage of C) mMDSC are shown. Differences in percentage or activation between mCRPC and HD were analyzed with the two-sample Mann-Whitney U test. Differences were considered significant when p<0.05, as indicated with the given p-value.
mMDSC
% cells
CRPC
w0v1
healthy
donors
