# Supplementary material for: Myeloid derived suppressor and dendritic cell subsets are related to clinical outcome in prostate cancer patients treated with prostate GVAX and ipilimumab
Source: J Immunother Cancer. 2014 Sep 16;2:31. doi: 10.1186/s40425-014-0031-3 (PMC4507359; doi:10.1186/s40425-014-0031-3)
Supplement: Additional file 4: Figure S4. — Differential leukocyte analysis in mCRPC patients before and during prostate GVAX/ipilimumab therapy. Absolute lymphocytes (white squares) and monocyte (black squares) counts were determined before (week 0/visit 1 (w0v1)), during (w4v3, w8v5, w16v9) and after (follow-up (fu)) prostate GVAX/ipilimumab therapy. Mean absolute counts ± SEM is given in 10e6 per ml of blood for lymphocytes (white squares), monocytes (black squares) and the sum of lymphocytes and monocytes (i.e. PBMC, grey squares). Differences between pre- and on- or post-treatment were analyzed with the repeated measures ANOVA with a post-hoc Dunnett’s multiple comparisons test. Differences were considered significant when p < 0.05, as indicated with an asterisk (* p < 0.05, ** p < 0.01). [file 40425_2014_31_MOESM4_ESM.ppt]

## Slide 1
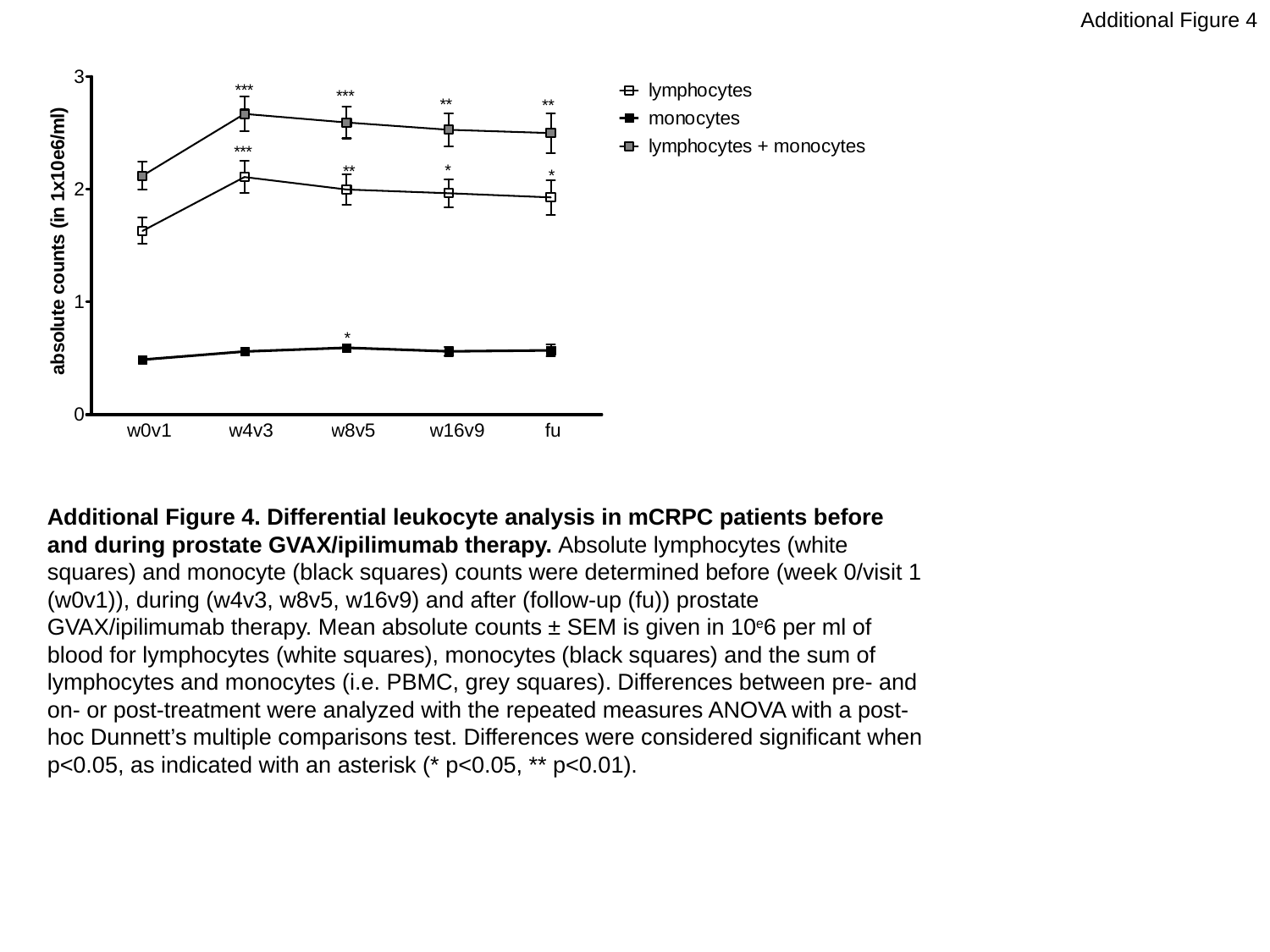

Additional Figure 4
Additional Figure 4. Differential leukocyte analysis in mCRPC patients before and during prostate GVAX/ipilimumab therapy. Absolute lymphocytes (white squares) and monocyte (black squares) counts were determined before (week 0/visit 1 (w0v1)), during (w4v3, w8v5, w16v9) and after (follow-up (fu)) prostate GVAX/ipilimumab therapy. Mean absolute counts ± SEM is given in 10e6 per ml of blood for lymphocytes (white squares), monocytes (black squares) and the sum of lymphocytes and monocytes (i.e. PBMC, grey squares). Differences between pre- and on- or post-treatment were analyzed with the repeated measures ANOVA with a post-hoc Dunnett’s multiple comparisons test. Differences were considered significant when p<0.05, as indicated with an asterisk (* p<0.05, ** p<0.01).
